# Cardiac device implantation and device usage in Fabry and hypertrophic cardiomyopathy

**DOI:** 10.1186/s13023-021-02133-4

**Published:** 2022-01-06

**Authors:** Ravi Vijapurapu, William Bradlow, Francisco Leyva, James C. Moon, Abbasin Zegard, Nigel Lewis, D. Kotecha, Ana Jovanovic, Derralynn A. Hughes, Peter Woolfson, Richard P. Steeds, Tarekegn Geberhiwot

**Affiliations:** 1 Department of Cardiology, Queen Elizabeth Hospital, Birmingham, UK; 2 Institute of Cardiovascular Sciences, University of Birmingham, Birmingham, UK; 3 Department of Endocrinology, Department of Inherited Metabolic Disorders, Queen Elizabeth Hospital, Edgbaston, Birmingham, B15 2TH UK; 4 Aston Medical Research Institute, Aston Medical School, Birmingham, UK; 5 Department of Cardiology, Barts Heart Centre, London, UK; 6 South Yorkshire Cardiothoracic Centre, Northern General Hospital, Sheffield, UK; 7 Mark Holland Metabolic Unit, Salford Royal Hospital, Salford, UK; 8 Lysosomal Storage Disorder Unit, Royal Free Hospital, London, UK; 9 Department of Cardiology, Salford Royal Hospital, Salford, UK; 10 Institute of Metabolism and System Research, University of Birmingham, Birmingham, UK

**Keywords:** Fabry, Hypertrophic cardiomyopathy, Arrhythmia, Defibrillator, Prognosis, Risk

## Abstract

**Background:**

Fabry disease (FD) is a treatable X-linked condition leading to progressive cardiac disease, arrhythmia and premature death. We aimed to increase awareness of the arrhythmogenicity of Fabry cardiomyopathy, by comparing device usage in patients with Fabry cardiomyopathy and sarcomeric HCM. All Fabry patients with an implantable cardioverter defibrillator (ICD) implanted in the UK over a 17 year period were included. A comparator group of HCM patients, with primary prevention ICD implantation, were captured from a regional registry database.

**Results:**

Indications for ICD in FD varied with 72% implanted for primary prevention based on multiple potential risk factors. In FD and HCM primary prevention devices, arrhythmia occurred more frequently in FD over shorter follow-up (HR 4.2, *p* < 0.001). VT requiring therapy was more common in FD (HR 4.5, *p* = 0.002). Immediate shock therapy for sustained VT was also more common (HR 2.5, *p* < 0.001). There was a greater burden of AF needing anticoagulation and NSVT in FD (AF: HR 6.2, *p* = 0.004, NSVT: HR 3.1, *p* < 0.001).

**Conclusion:**

This study demonstrates arrhythmia burden and ICD usage in FD is high, suggesting that Fabry cardiomyopathy may be more ‘arrhythmogenic’ than previously thought. Existing risk models cannot be mutually applicable and further research is needed to provide clarity in managing Fabry patients with cardiac involvement.

## Background

Fabry disease (FD) is an X-linked lysosomal storage disorder with a deficiency in the enzyme α-Galactosidase A. Cardiovascular complications are a cardinal feature and include progressive left ventricular hypertrophy (LVH), myocardial inflammation, fibrosis, arrhythmia, congestive cardiac failure and sudden death. Sphingolipid deposition occurs in all cardiac cells [1], leading to a cascade of cellular reactions producing a pro-inflammatory microenvironment within cardiac myocytes, conduction tissue injury and apoptosis, contributing to electrical instability. Although inherited in an autosomal dominant pattern with different pathogenesis based on sarcomeric gene variants, hypertrophic cardiomyopathy (HCM) is the paradigm hypertrophic cardiomyopathy and is perceived to be one of the heart muscle disorders at most risk of malignant arrhythmia. This study aimed to increase awareness of Fabry cardiomyopathy amongst clinicians and highlight the potential for arrhythmogenicity, by comparing the frequency of device usage in patients with Fabry cardiomyopathy and sarcomeric HCM.

## Methods

This is a multi-center, retrospective sub-study of all Fabry patients within the UK who had an implantable cardioverter defibrillator (ICD) inserted between 1st December 2000 and 1st February 2018 (n = 50, 80% males, 51% non-classical mutation, mean age at device implantation 57 ± 12 years). This cohort was taken from a larger study evaluating Fabry patients with any cardiac device implanted, where detailed demographic data can be seen [2]. A comparator group included 64 age-matched HCM patients with an ICD implanted for primary prevention captured from a regional registry database (67% males, all gene positive, mean age at implant 56 ± 19 years). Local clinical governance approval was obtained. Cardiac investigations (12-lead electrocardiograms [ECGs] and cardiac magnetic resonance imaging [CMR]) were collected if performed before or within 3 months of device implantation. ECG abnormalities included prolonged/shortened PR interval, QRS duration > 120 ms, minor conduction disturbances (intraventricular conduction abnormalities and bundle branch block patterns < 120 ms), the presence of LVH by Sokolow-Lyon criteria, T wave inversion in at least two contiguous leads and the presence of multifocal ventricular ectopy. Arrhythmias were identified from ICD follow-up reports, which included: atrial fibrillation (AF) needing anticoagulation, non-sustained ventricular tachycardia (NSVT; defined as 3 or more ventricular beats at a rate > 120 bpm for less than 30 s) and sustained VT (rate > 120 bpm for more than 30 s) or ventricular fibrillation requiring ICD therapy.

Statistical analyses were carried out using SPSS 23 (IBM, Armonk, NY). All continuous variables are expressed as mean ± standard deviation and all non-continuous data are expressed as frequencies or percentages. Normality was evaluated using the Shapiro–Wilk test and with visual inspection of the data. Groups were compared with independent t-testing for parametric data and Mann–Whitney U testing for non-parametric data. Chi-squared testing with Yates correction or Fishers-exact testing (for comparisons with less than 5 occurrences) was used to compare proportions within two independent groups. Time-to-event analysis was performed to evaluate the presence of arrhythmic events. Kaplan–Meier curves were utilized to show time to first arrhythmia, first new diagnosis of AF and first appropriate ICD therapy, whereas a multivariable Cox model was used for other parameters. Proportionality of hazards was assessed by visual inspection of Kaplan–Meier curves for each predictor variable. A *p* value of < 0.05 was considered statistically significant.

## Results

Fifty FD patients with an ICD implanted were compared to 64 age- and gender-matched HCM patients. Twenty eight percent of ICDs in FD were implanted for secondary prevention following a symptomatic ventricular arrhythmia. The remaining 72% were for primary prevention following electrophysiology multi-disciplinary team meeting in context of multiple potential risk factors and other coexisting high-risk diagnoses. Table 1 highlights the factors considered to confer high arrhythmic risk from multidisciplinary team assessment.

**Table 1:** Proportion of arrhythmic risk factors in Fabry cohorts

	Fabry disease		
	Primary prevention N = 36	Secondary prevention N = 14	*p* value
Age (years)	58 ± 12	57 ± 12	0.922
Male gender (n, %)	8 (22)	2 (7)	0.704
MSSI > 20 (n, %)	10 (28)	2 (14)	0.468
LVH (n, %)	32 (89)	12 (86)	1.000
LGE > 3 segments (n, %)	11 (41)	3 (11)	0.115
Elevated troponin (n, %)	7 (19)	4 (29)	0.476
QRS duration > 120 ms (n, %)	21 (58)	7 (50)	0.719

Fabry patients who underwent secondary prevention ICD implantation, tended to have clinical parameters suggestive of more advanced disease. Baseline indexed LV mass on CMR was higher in devices implanted for secondary prevention (primary prevention: 143.6 ± 38.4 vs. secondary prevention: 164.0 ± 45.0, *p* = 0.379), but the extent of late gadolinium enhancement (LGE) was greater in those who underwent device implantation for primary prevention purposes (primary prevention: 14/16, 88% vs. secondary prevention: 5/9, 56%, *p* = 0.142), although neither reached statistical significance due to low numbers.

### Fabry disease versus hypertrophic cardiomyopathy

All HCM patients were risk stratified and underwent device implantation for primary prevention based on an estimated European Society of Cardiology (ESC) 5-year risk of sudden cardiac death (SCD) greater than 4%. All comparisons between FD and HCM were of patients who underwent device implantation for primary prevention only.

While recognizing that the pathogenesis of cardiac involvement in FD and HCM cohorts is different and therefore that disease phenotypes may be different, comparison of established risk factors for SCD risk stratification revealed higher indexed LV mass on CMR in FD compared to HCM (144 ± 38 g/m^2^ vs. 102 ± 36 g/m^2^, *p* = 0.009) [3]. Maximum wall thickness (MWT) thickness, however, was similar in both groups (FD: 21.7 ± 5.5 vs. HCM: 21.6 ± 4.7, *p*-0.972). The proportion of patients with asymptomatic NSVT on Holter monitoring prior to device implantation was higher in FD, but this was not significant (10/36, 28% vs. 11/64, 17%, *p* = 0.321). There was a tendency to a reduced LV function in Fabry patients (LVEF < 50% on echocardiography—FD: 10/36, 28% vs. HCM: 7/64, 11%, *p* = 0.061). The degree of late gadolinium enhancement (LGE) on CMR was similar in both cohorts (FD: 14/16, 88% vs. HCM: 25/28, 89%, *p* = 1.000). Although no change in PR interval was observed, QRS duration was greater in FD (135 ± 32 ms vs. 116 ± 30 ms, *p* = 0.006). FD patients had greater high-sensitive (hs) troponin I (121 vs. 19 ng/l, *p* < 0.001) but a non-significant trend toward higher NT-pro-BNP (1708 vs. 888 ng/l, *p* = 0.086). Comparison of other risk factors such as syncope or a family history of sudden death was not possible due to a lack of historical data documented in the FD cohort. Detailed demographic and investigation data can be seen in Table 2.

**Table 2:** Clinical demographics and investigation data: Fabry versus HCM

	Fabry			HCM: primary prevention	*p* value^c^
	Primary prevention	Secondary prevention	*p* value^b^		
Sample size (n, %)	36 (72)	14 (28)	*N/A*	64	*N/A*
Follow-up duration (years)	3.8 ± 2.6	5.8 ± 3.9	*0.036*	6.4 ± 2.9	< *0.001*
Age (years)	58 ± 12	57 ± 12	*0.922*	56 ± 19	*0.516*
Male gender (n, %)	8 (16)	2 (4)	*0.704*	21 (33)	*0.373*
On ERT (n, %)	23 (46)	11 (22)	*0.501*	–	*N/A*
Classical mutation (n, %)	16 (32)	4 (8)	*0.353*	–	*N/A*
BMI (kg/m^2^)	27.0 ± 6.0	31.6 ± 7.2	*0.087*	28.4 ± 6.2	*0.411*
HR (bpm)	64 ± 16	55 ± 8	*0.265*	66 ± 12	*0.633*
SBP (mmHg)	122 ± 22	124 ± 19	*0.824*	128 ± 22	*0.436*
DBP (mmHg)	71 ± 16	73 ± 7	*0.785*	76 ± 12	*0.292*
MSSI	16.7 ± 9.4	11.9 ± 7.1	*0.104*	–	*N/A*
*Comorbidities*					
IHD (n, %)	2 (4)	0 (0)	*1.000*	3 (5)	*1.000*
CKD stage 3–5 (n, %)	8 (16)	0 (0)	*0.087*	0 (0)	**< *0.005***
HTN (n, %)	8 (16)	2 (4)	*0.704*	16 (25)	*0.946*
DM (n, %)	3 (6)	2 (4)	*0.611*	4 (6)	*0.700*
Stroke/TIA (n, %)	7 (14)	0 (0)	*0.169*	2 (3)	***0.010***
*ECG*	n = 48			n = 53	
Abnormal (n, %)	33 (69)	13 (27)	*1.000*	43 (81)	*0.114*
AF/PAF (n, %)	3 (6)	0 (0)	*0.550*	3 (6)	*0.664*
PR interval (ms)	172 ± 36	145 ± 30	*0.051*	178 ± 39	*0.557*
QRS duration (ms)	135 ± 32	134 ± 32	*0.939*	116 ± 31	***0.006***
*Echocardiography*	n = 48			n = 62	
LVH (n, %)	32 (67)	12 (25)	*1.000*	55 (89)	*0.485*
LA dilated (n, %)	24 (50)	10 (21)	*1.000*	30 (48)	*0.060*
*CMR*	n = 27			n = 28	
LVEDV (ml)	158.2 ± 75.0	143.0 ± 18.3	*0.791*	145.0 ± 53.2	*0.564*
LVESV (ml)	73.0 ± 80.3	26.0	*0.594*	54.5 ± 44.9	*0.386*
LVEF (%)^a^	58 (53–65)	55 (43–65)	*0.747*	57 (55–64)	*0.904*
LVMi (g/m^2^)	143.6 ± 38.4	164.0 ± 45.0	*0.379*	102.4 ± 35.7	***0.009***
MWT (mm)	21.8 ± 5.2	21.3 ± 4.0	*0.867*	21.6 ± 5.5	*0.965*
LGE (n, %)^d^	14/16 (88)	5/9 (56)	*0.072*	25 (89)	*1.000*
Extensive (> 3 AHA segments)	*11/16 (69)*	3/9 (33)	*0.115*	*14 (50)*	*0.373*
Mild (1–2 AHA segment e.g. BIFL)	*3/16 (19)*	2/9 (22)	*1.000*	*9 (32)*	*0.487*
RV insertion point	*0 (0)*	0 (0)	*N/A*	*2 (7)*	*0.526*
*Biomarkers*					
High sensitive troponin T (ng/L)^a^	121 (51–154)	90 (44–272)	*0.927*	19 (13–38)	** < *0.001***
NT-pro BNP (ng/l)^a^	1708 (626–4068)	1319 (719–1894)	*0.667*	888 (353–2070)	*0.081*

Bold italics indicate statistically significant results (*p* < 0.05)

Italics indicate the level of significance

^a^Non-parametric data so presented as median (IQR)

^b^*p* value comparing primary and secondary prevention device implantation in FD

^c^*p* value comparing primary prevention device implantation in FD and HCM

^d^Proportions taken from patients who had a CMR and were given Gadolinium contrast

### Assessment of arrhythmia

#### Fabry disease: primary versus secondary prevention devices

The occurrence of any arrhythmia requiring treatment as a combined endpoint or AF alone as a single endpoint tended to be higher in Fabry patients who had a device implanted for secondary prevention, although this did not reach statistical significance due to low numbers (any arrhythmia: primary—17/36, 47% vs. secondary—10/14, 71%, *p* = 0.206 and AF: primary—7/36, 19% vs. secondary—4/14, 29%, *p* = 0.476). VT requiring immediate shock therapy however, occurred more frequently in those with a secondary prevention device (hazard ration [HR] 2.7, 95% confidence interval [CI] 1.2–5.7, *p* < 0.001). Of the 14 Fabry patients with ventricular arrhythmia requiring ICD therapy (anti-tachycardia pacing [ATP] ± defibrillation), 10 had devices for primary prevention and 4 for secondary prevention. Within the primary prevention cohort, 6 patients were treated with ATP alone and 4 patients with ATP and subsequent defibrillation. All patients within the secondary prevention cohort required defibrillation. All therapies were for sustained monomorphic VT.

#### Fabry disease versus hypertrophic cardiomyopathy: primary prevention devices only

**Fig. 1:**
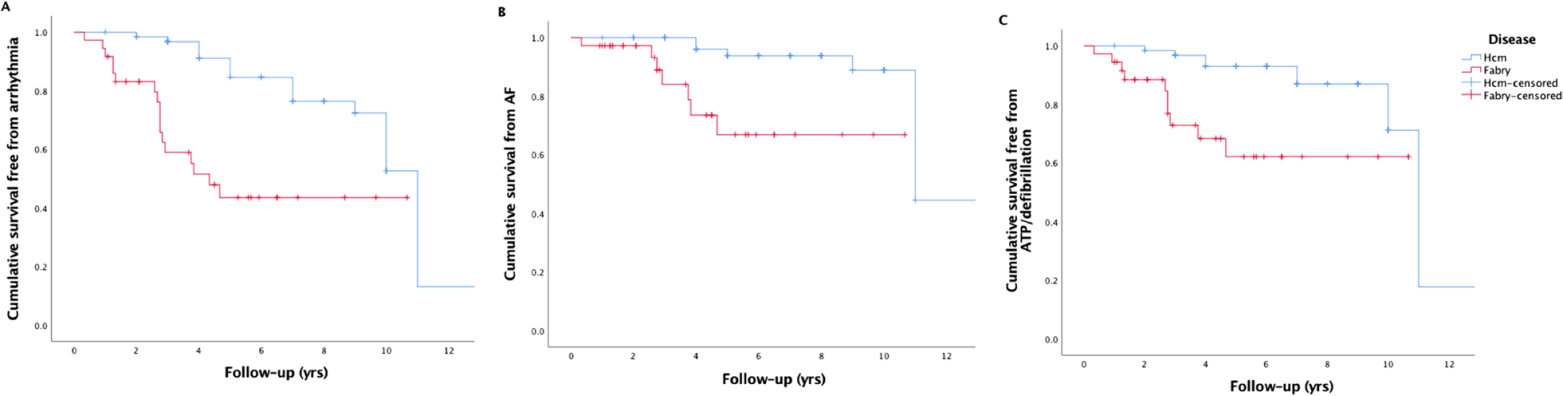
Survival free of any arrhythmic event, atrial fibrillation and ventricular arrhythmia requiring ATP/defibrillation therapy, in Fabry and hypertrophic cardiomyopathy. Event rates in Fabry and HCM. **A** Time to first arrhythmic event (*p* < 0.001). **B** Time to first episode of AF requiring anticoagulation (*p* = 0.001). **C** Time to first appropriate ATP/defibrillation therapy (*p* < 0.001)

When evaluating FD and HCM primary prevention devices, arrhythmia occurred more frequently in FD over shorter follow-up (HR 4.2, 95% CI 2.0–8.6, *p* < 0.001). VT requiring ATP ± defibrillation therapy was more common in the Fabry cohort (HR 4.5, 95% CI 1.7–11.7, *p* = 0.002, see Fig. 1C). Shock therapy for sustained VT was also more common in FD (HR 2.5, 95% CI 1.6–3.9, *p* < 0.001). There was a greater burden of AF needing anticoagulation and NSVT in FD compared with HCM (AF: HR 6.2, 95% CI 1.8–21.6, *p* = 0.004, NSVT: HR 3.1, 95% CI 1.7–5.6, *p* < 0.001). FD was also found to be an independent predictor of all arrhythmia types in multivariate Cox regression with age and gender. FD patients who had arrhythmia were often older, had greater LV mass, more scar tissue, a larger left atrium and a broader QRS duration. This did not however, reach statistical significance possibly due to low numbers.

## Discussion

This study has shown that ICD usage related to the burden of arrhythmia in secondary prevention devices was higher in FD than age and sex-matched controls with HCM, with a notably greater frequency of VT requiring immediate shock therapy. Patients with FD had a higher frequency of ventricular arrhythmia requiring ATP/defibrillation therapy. Furthermore, although the indication for ICD implantation in FD was variable and often unclear, device delivered therapy was more frequent in FD compared to guideline directed device use in HCM. This may reflect the fact that risk prediction calculators specifically exclude use in FD patients, and no equivalent is available for FD. Despite this, standard arrhythmic risk factors used to guide ICD implantation in HCM occurred more frequently in the FD population than in age and sex-matched HCM patients, and their presence in this group may be associated with malignant ventricular arrhythmia.

Although the precise mechanisms of arrhythmia are not fully understood, there are similarities between FD and HCM in terms of the structural changes that predispose to VA and sudden cardiac death, including left ventricular hypertrophy, ventricular dysfunction and extensive fibrosis with myocardial scarring [4] that suggest early therapeutic intervention may be beneficial [5]. The incidence of sustained VA requiring device therapy was higher however, in the FD cohort compared to both the HCM cohort and previous studies of subjects with non-ischemic cardiomyopathy [6], thus suggesting that device implantation in FD is delayed until the disease is more advanced or complex than in the other cohorts. It is also possible that Fabry cardiomyopathy is more ‘arrhythmogenic’ and consequently risk models between disease processes cannot be mutually applicable. Further research is needed, as such a finding may have significant implications on future monitoring and treatment in FD patients with cardiac involvement [7].

The limitations of this study include that the burden of arrhythmia may have been underestimated, with specific arrhythmias such as slow VT that are not within the device detection zone being overlooked. Additionally direct phenotypic comparisons between FD and HCM were difficult as FD is a rare disease and matching for age, gender and disease severity (LVMi) was not possible due to low patient numbers.

## Conclusion

Arrhythmia burden and ICD usage in Fabry is high, suggesting that Fabry cardiomyopathy is more ‘arrhythmogenic’ than previously thought and further research is needed to define the risk profile in greater detail.

## Data Availability

The datasets used and/or analyzed during the current study are available from the corresponding author on reasonable request.

## Abbreviations

FD: Fabry disease
LVH: Left ventricular hypertrophy
HCM: Hypertrophic cardiomyopathy
ICD: Implantable cardioverter defibrillator
ECG: Electrocardiogram
CMR: Cardiac magnetic resonance imaging
LVH: Left ventricular hypertrophy
AF: Atrial fibrillation
NSVT: Non-sustained ventricular tachycardia
ESC: European Society of Cardiology
SCD: Sudden cardiac death
LGE: Late gadolinium enhancement
MWT: Maximum wall thickness
LVEF: Left ventricular ejection fraction
HR: Hazard ratio
CI: Confidence interval
